# Why labelling the ‘fast-reacting thiols’ of myosin reduces force output: re-assessing a year at the University of California San Francisco (1987-8)

**DOI:** 10.1007/s12551-026-01425-y

**Published:** 2026-02-27

**Authors:** Glenn Carrington, Michelle Peckham

**Affiliations:** Faculty of Biological Sciences and the Astbury Centre for Structural Biology, University of Leeds, Leeds, UK

**Keywords:** Myosin, IATR, Muscle contraction, Fluorescence polarisation

## Abstract

The fast-reacting thiol in the SH1 helix of myosin was often used to attach spin or fluorescent probes to the motor domain, to readout myosin orientation during the 1980s. However, at this time, we found that labelling this residue (Cys707) in myosin in skinned rabbit psoas fibres, using iodoacetacetamidotetramethyl rhodamine (IATR), reduced force output during active contraction. With access to recent pre- and post-powerstroke actomyosin structures, we can now explain these results. We modelled IATR onto the equivalent SH1 cysteine residue in the pre-power and post-power structures of myosin 5. This revealed that labelling of the fast-reacting thiol would interfere with the structural changes in the relay and SH1 helices required to generate the power stroke, explaining why force output reduces.

As is well established, muscle contraction is driven by the interaction of the molecular motor myosin with actin, fuelled by ATP hydrolysis. Myosin is organised into thick filaments, 1.6 µm long along with myosin binding-protein C and titin. Actin is organised into thin filaments, ~ 1.0 m long, along with troponin and tropomyosin, and in skeletal muscle nebulin is also present. The filaments are organised into repeating units (muscle sarcomeres), bounded by Z-discs, which are about 2–2.5 µm long, depending on the muscle type. Sarcomeres are arranged end-to-end along the length of myofibrils, which run along the length of a muscle fibre, such that a small shortening of each sarcomere adds up in series, to generate a large movement at the ends of the muscle fibre. We now understand many of the details about the structure of the motor in different states of the ATPase cycle, both attached and detached from actin (reviewed in Irving 2025; McMillan and Scarff 2022; Robert-Paganin et al. 2020; Wang and Raunser 2023; Wang and Raunser 2023). We have also established that in relaxed muscle (high Mg.ATP, low Ca^2+^), many of the myosin heads are organised in what is termed the ‘interacting heads’ motif, as most recently shown by CryoEM Tomography (Dutta et al. 2023; Tamborrini et al. 2023). In this state, the two heads interact, and ATP usage is low, first coined by Roger Cooke as the ‘super-relaxed’ (SRX) state (Stewart et al. 2010).

In 1987, the field did not have a structure for myosin or actin. The atomic structure for globular (G-) actin was not solved until 1990 (Kabsch et al. 1990), and that for the myosin in 1993 (Rayment et al. 1993). To try to understand how myosin interacted with actin or changed its conformation, many indirect approaches were used. At the time, one of the authors (Michelle) was working as a research fellow with Malcolm Irving in the MRC Biophysics Unit at Kings College London, and she used muscle birefringence, a label-free technique sensitive to cross-bridge orientation, to investigate cross-bridge orientation in relaxed (+ Mg.ATP) and rigor (-Mg.ATP) muscle. Using this approach, she discovered that in relaxed fibres, the long axis of the heads appears to be relatively well aligned with the filament axis, consistent with what we now know the structure to be in relaxed muscle (Peckham and Irving 1989). She presented this work at a Biophysics conference in San Francisco in 1986; a nerve-wracking 10-min talk with 10 min of questions. These findings were relatively controversial at the time; many other groups had been using either fluorescence or spin labels attached to the cysteine residue in the SH1 helix of the myosin motor to investigate myosin orientation, and they showed that the myosin orientation was disordered (Thomas 1987; Thomas and Cooke 1980).

Michelle then moved to San Francisco to the Cardiovascular Research Institute (CVRI) at the University of California, San Francisco (UCSF) to work in the Group of Manuel Morales. Her project was to measure changes in the myosin cross-bridge (myosin head) orientation during contraction, or fast ATP release from caged ATP using fluorescence polarisation. Up until that point, the Morales group had been labelling the reactive cysteine residue (Burghardt et al. 1984) to perform fluorescence polarisation measurements, but had not generally measured active contraction. Michelle has also just investigated changes to birefringence following the release of Mg.ATP using caged ATP to activate muscle fibres from rigor (Peckham et al. 1994). The Morales group seemed keen to use this approach for fluorescence polarisation measurements. That goal was never achieved because the lab was not equipped either with the laser required to do this (at the time, a frequency doubled dye laser from Candela), giving a pulse of light at 320 nm, or a Xenon flash lamp developed to do the same. However, Michelle set up a method to measure force from single muscle fibres during contraction while measuring fluorescence polarisation.

Michelle prepared single skinned rabbit psoas fibres, in the same way that she had used for birefringence experiments (Peckham et al. 1994; Peckham and Irving 1989). Solutions had a final ionic strength of 100 mM and substituted propionate instead of chloride. This avoids changes in lattice spacing when switching between relaxed, rigor and contracting solutions. Fibres were labelled using iodoacetacetamidotetramethyl rhodamine (IATR) obtained from a company (Molecular Probes, now owned by ThermoFisher). This reagent predominantly labels the Cys707 in skeletal myosin (also known as the highly reactive thiol SH1 (Rayment and Holden 1994)), and labelling was done using the standard techniques of the lab (Borejdo et al. 1979). The fibres were labelled with a range of IATR from 0 to 400 µM in a relaxing solution, and then washed to remove excess label. The fibres were then either switched into rigor solution (as relaxing solution, but no Mg.ATP), or activating solution (as relaxing solution, but with the addition of Ca^2+^) (Peckham et al. 1990). After measuring force (Fig. 1A) and fluorescence polarisation, a highly capable undergraduate student (Katie) analysed the fibres by SDS gel electrophoresis and used fluorescence imaging to confirm that the myosin heavy chain had been labelled (Fig. 1A).

**Fig. 1:**
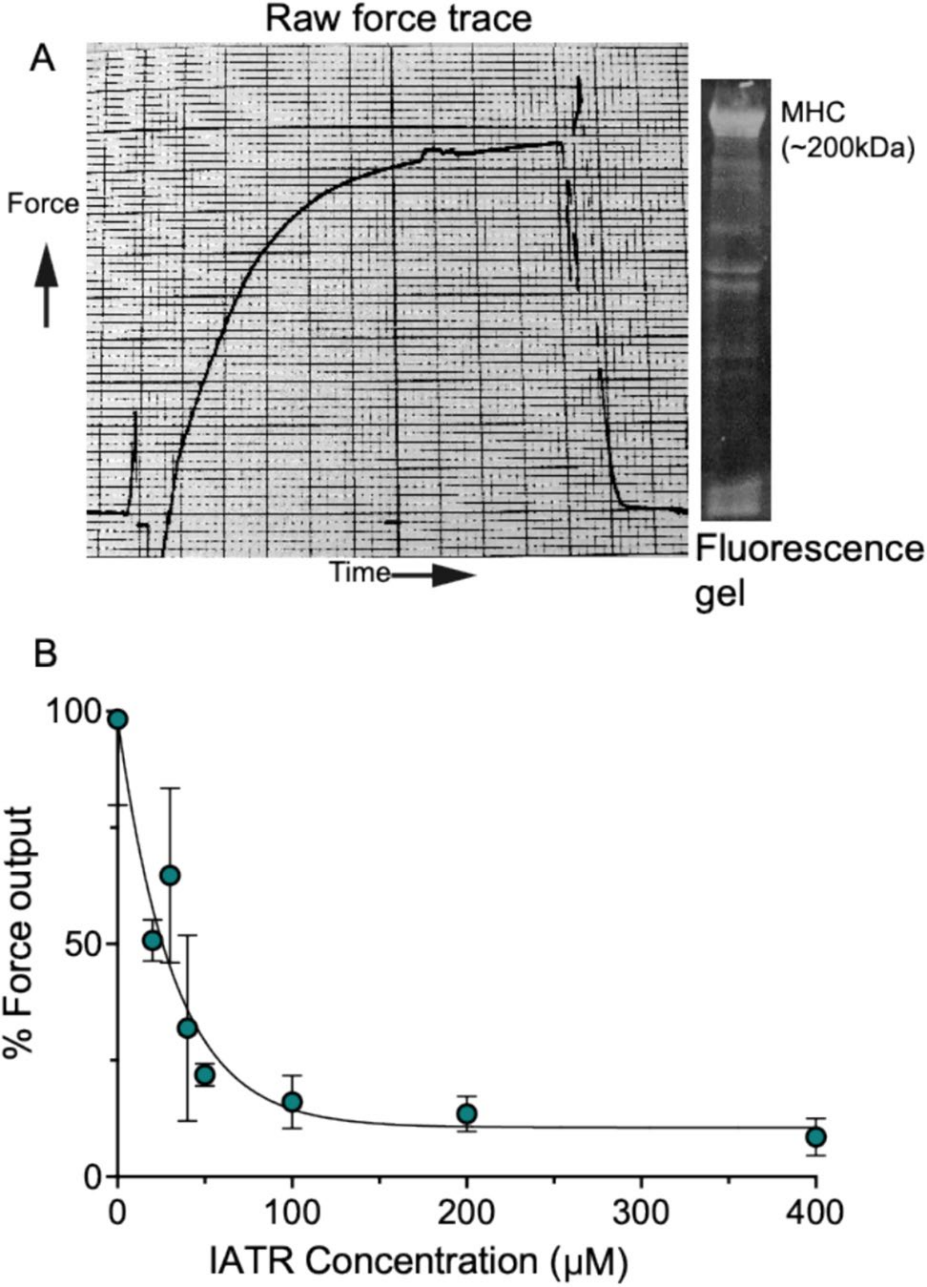
Testing the effect of IATR labelling on force generation. **A** Single glycerinated rabbit psoas fibres, labelled with IATR generated force when the solution was changed from relaxing to activating solution. IATR labelling was confirmed by SDS PAGE of the samples after this analysis and using fluorescence to identify that it was predominantly the myosin heavy chain (MHC) that was labelled. **B** Results from at least 3 separate experiments to test the effect of lATR labelling on the relative force output. Mean ± S.D. is shown for each of the values. Curve fitted using Prizm (GraphPad)

Michelle discovered two things from these experiments. One was that the force generated decreased as the concentration of label increased (Fig. 1B). The other was that she could not detect any significant change in fluorescence polarisation. One possibility for not observing a change in fluorescence polarisation was that the commercial IATR may have contained two isomers, although a subsequent study seems to have ruled this out (Berger et al. 1996). The effects on force were, however, observed by other research groups (Berger et al. 1996). However, without a structure, she could not uncover the underlying mechanism for these observations, and this data was never published.

Now, a wide range of structures for myosin and for actomyosin have been published. Given the benefit of this new structural understanding of how myosin generates force, can we now understand why labelling myosin with IATR is so disruptive and may inhibit force generation? Recently, the structure of the pre-power stroke of myosin attached to actin was solved using myosin 5a (Klebl et al. 2025). Myosin 5a has a serine and not a cysteine residue in the SH1 helix (Fig. 2A). However, modelling the IATR into this structure shows how it lies between the relay helix and the SH1 helix and would inhibit the changes to the structure of these helices critical for the power stroke, following Pi release (Fig. 2B). Labelling a second cysteine in this region would also likely be disruptive. Thus, Michelle’s original data obtained many years ago was probably correct: labelling with IATR interferes with force generation. Fortunately, despite not publishing her findings, this effect of labelling the reactive thiol gradually became clear to the field and has not now been used for some time.

**Fig. 2:**
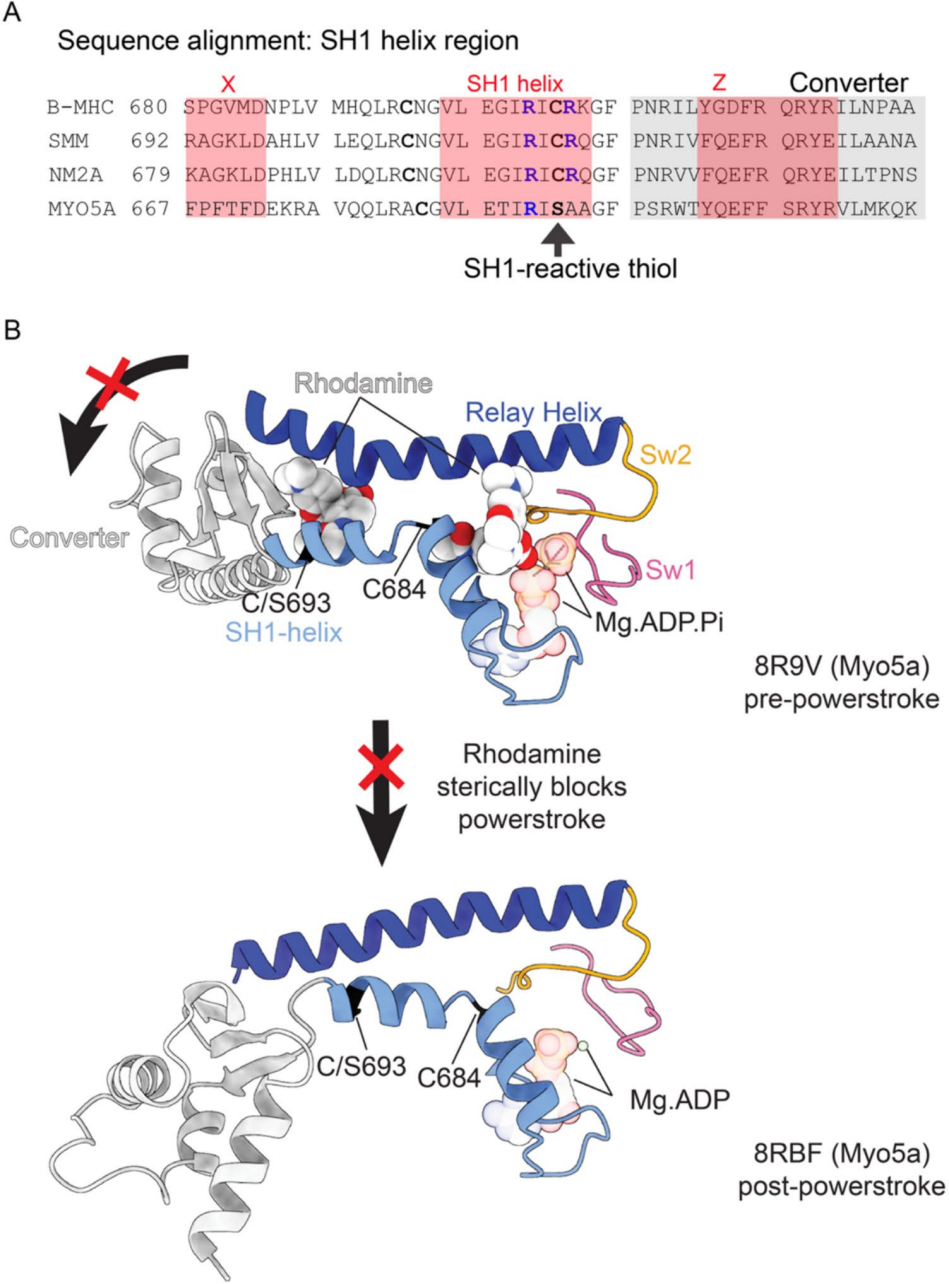
**A** Sequence alignment of β-cardiac myosin heavy chain (β—MHC), smooth muscle myosin (SMM), non-muscle myosin 2 A (NM2A) and myosin 5a (Myo5a) to show the positions of the highly reactive thiol in the SH1 helix (substituted to serine in Myo5a), together with a second cysteine residue, termed SH2, which can also be labelled, although the SH1 thiol is most reactive. **B **The SH1 thiol (C/S693) lies between the relax helix and the SH1 helix and labelling of this residue with IATR would likely block the power stroke, as would labelling of the SH2 thiol (C684)

Where does Roger Cooke come into all of this? Michelle first met Roger at the Biophysics meeting in San Francisco in 1986. Roger was well known for hosting a party at his wonderful house on Willard Street, a short walk away from UCSF on Parnassus Avenue. Just before she arrived in San Francisco to work with Manuel Morales the following year, she met him again at the Gordon Conference in Tilton on Muscle: Contractile Proteins in August 1987. Roger was just about to go on holiday for 2 weeks after the meeting and amazingly he handed over the keys to his house and said she could stay there while she looked for her own place to live (at least until he was back). Just one example of his incredible generosity. Roger had in fact also worked as a postdoctoral fellow in Manuel Morales’ lab but by then had his own independent research group at UCSF. In his group was another English postdoc (Anthony Baker), who also kindly helped Michelle get settled. Roger and Michelle played tennis regularly and occasionally went dancing (Marenge!). When she told him about my issues in the lab, and the disbelief at my findings, he was very supportive. Eventually Michelle left to work in David White’s lab in September 1988, at the University of York, before starting her Royal Society Fellowship at the Biophysics unit at King's College London in 1990.

Roger was a great friend and colleague, here pictured (Fig. 3) at Michelle’s leaving party (from San Francisco), together with Anthony Baker. He made a wide range of important contributions to the field, and his Biophysical Society parties at his house were legendary. Michelle very much appreciated his help and support not just while in San Francisco, but for many years afterwards.

**Fig. 3:**
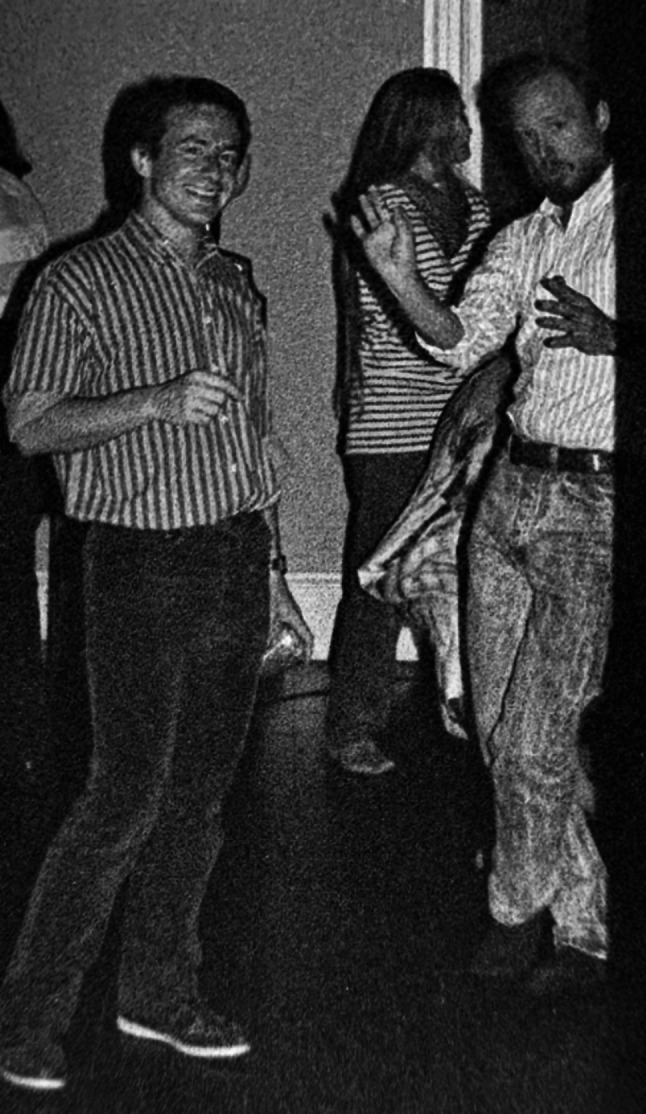
Anthony Baker (LHS) and Roger Cooke (RHS) enjoying themselves at Michelle’s leaving party in San Francisco in 1988

## Data Availability

No datasets were generated or analysed during the current study.
